# The Structure of American Political Discontent

**DOI:** 10.1093/poq/nfac009

**Published:** 2022-04-29

**Authors:** Jack Santucci, Joshua J Dyck

**Affiliations:** assistant teaching professor in the Department of Politics at Drexel University, Philadelphia, PA, USA; professor in the Department of Political Science at the University of Massachusetts Lowell, Lowell, MA, USA

## Abstract

We explore the role of “political discontent” as a second dimension of American public opinion. Others have shown that a second dimension tends to capture social and/or racial attitudes. What happens when indicators of discontent are included in such analyses? Using data from two surveys and the ordered optimal classification (OOC) procedure, we scale seven items from the “discontent” literature alongside a larger set of questions that has been shown to capture the two-dimensional structure of mass opinion. Discontent items dominate the second dimension in both data sets. Further, five of the seven items predict voting for “insurgents” in the 2016 presidential primaries. Second-dimension attitudes matter in elections and concern the political system writ large. By extension, the liberal-conservative heuristic gives an incomplete picture of mass political behavior.

Typical understandings of American politics rest on a liberal-conservative heuristic. When empirical data are inconsistent with this unidimensional presumption, some give evidence that the public is non ideological (Kinder and Kalmoe 2017). Others argue that ideology is structured by *two dimensions*, at one time labeled “economic” and “social” (Schofield, Miller, and Martin 2003; Treier and Hillygus 2009). Yet several have noted the recent salience of attitudes about “the system” or “establishment” (e.g., Drutman 2016; Buisseret and Van Weelden 2020, p. 359). This note explores the role of such attitudes in defining public opinion’s second dimension.

We project two sets of measures into the space of American mass opinion as of 2016. While these measures have pedigree in other literatures—namely on “stealth democracy” (Hibbing and Theiss-Morse 2002) and “external efficacy” (Craig 1980; Craig, Niemi, and Silver 1990)—they frequently appear in work on “political discontent” (Rooduijn, van der Brug, and de Lange 2016; Jennings et al. 2017) or “process” attitudes (Coffé and Michels 2014; Hibbing et al. 2021). A typical study compares such items to each other, then sees whether an index predicts anti-establishment vote choice (Akkerman, Mudde, and Zaslove 2014; Rooduijn, van der Brug, and de Lange 2016; Spruyt, Keppens, and Van Droogenbroeck 2016; Geurkink et al. 2020; Hibbing et al. 2021). Our approach differs by comparing “discontent” items to several others that capture the wider opinion structure. We use a nonparametric scaling procedure to make these comparisons.^1^

If discontent attitudes constitute the second ideological dimension and matter for voting, it follows that a liberal-conservative perspective misses an important aspect (indeed, a dimension) of political behavior. Our results suggest that discontent attitudes are important. First, we find that five of our seven items of interest do more to define the second dimension than any other item in the data—including those previously shown to define it as “social.” Second, we show that several of these items are individually correlated with Trump and Sanders support in the 2016 primaries. Namely, three of four stealth-democracy items predict Trump support, one external-efficacy item predicts Sanders support (“people like me have no say”), and one external-efficacy item predicts voting for both (“elites don’t understand”).

Our paper begins with additional justification for viewing discontent (or contentment) as orthogonal to liberal-conservative. Then, after discussing our methods and results, we conclude with how our findings relate to “populism” research comparatively and in the United States.

## Additional Justification for Viewing “Discontent” as Orthogonal to Liberal-Conservative

This note originates from two observations about public opinion. One is that American mass opinion has at least two dimensions, contra to the common view that people are liberal, conservative, or moderate. Several studies have shown this with a range of research methods (e.g., Schofield, Miller, and Martin 2003; Treier and Hillygus 2009). Often, the dimensions are labeled social/racial and economic, although Menchaca (2021) finds evidence for a third “morality” dimension.

The second observation is that theories of political discontent imply two-dimensional spaces of their own. Lipset’s (1959) account of “working-class authoritarianism” starts to go in this direction, positing separateness of attitudes toward redistribution and procedural democracy. Axelrod (1967, p. 57) continues in this vein, finding an attitude cluster he associates with 1890s populism: anti-civil liberties, anti-foreign intervention, and pro-social services. More recently, the “stealth democracy” hypothesis holds that views about *what* government should do might be independent of views on *how* government should do it. Evidence at the time included stealth-democratic attitudes among Ross Perot supporters and that the respective index was uncorrelated with five-point ideology (Hibbing and Theiss-Morse 2002). Finally, a newer theory of “ideational populism” casts anti-system attitudes as compatible with either socialism or ethnic nationalism (Mudde and Rovira Kaltwasser 2017).

One might then ask how a two-dimensional view of political discontent fits with the two-dimensional space of American politics. To answer that question, we use a nonparametric scaling procedure.

## Data and Methods

Our data come from two surveys, each including several items with “standard” liberal-conservative content. The first data source is a 1,000-respondent module of the 2016 Cooperative Congressional Election Study (CCES) (Gronke 2019),^2^ which included four common stealth-democracy items (VanderMolen 2017; Lavezzolo and Ramiro 2018):

1. Elected officials would help the country more if they would stop talking and just take action on important problems.
2. What people call “compromise” in politics is really just selling out on one’s principles.
3. Our government would run better if decisions were left up to non-elected, independent experts rather than politicians or the people.
4. Our government would run better if decisions were left up to successful business people.

The second data source is the Voter Study Group’s 2016 Views of the Electorate Research survey (or VSG) (Voter Study Group 2017).^3^ This had 8,000 respondents. It included two external-efficacy items studied by populism scholars (Rooduijn, van der Brug, and de Lange 2016, p. 36; Geurkink et al. 2020, p. 254), then a third validated by Craig, Niemi, and Silver (1990, pp. 299–300). These are, in order:

1. People like me don’t have any say in what the government does.
2. Elites in this country don’t understand the problems I am facing.
3. Elections today don’t matter; things stay the same no matter who we vote in.

To capture the two-dimensional space of American mass politics, we drew on Santucci (2020), who used 19 VSG items to test for spatial realignment. Factor analysis revealed two dimensions, termed “liberal-conservative” and “ethnoracial resentment.”^4^ These items cover race relations, immigration, climate, abortion, same-sex marriage, health policy, criminal punishment, trade, taxation, and regulation of business (see Supplementary Material, section III). From the CCES, we drew 18 items that were *as similar as possible* to the VSG items (see Supplementary Material, section II). One notable difference concerns the wealth tax, for which there was no CCES analogue. Instead, we included a ranking item on raising taxes,^5^ then constructed a four-category income/sales-tax trade-off variable. Another notable difference was CCES’s use of a new racism battery, which may behave differently from the “racial resentment” battery (DeSante and Smith 2020). Finally, the CCES did not include items on affirmative action nor business regulation. The two sets are similar, if not perfectly comparable,^6^ and we will see that each produces a credible image of the party system. We coded the external-efficacy and stealth-democracy items so that greater values reflect agreement. All other items were coded in the conservative direction.

To scale the items, we use Hare, Liu, and Lupton’s (2018) ordered optimal classification (OOC) procedure. This is similar to the NOMINATE “tool” used to analyze Congressional roll-call voting. As such, it produces figures of political cleavages: points giving people’s “ideological” locations, then lines that divide them on specific issues. It also produces measures analogous to factor loadings.

More technically, OOC orders respondents and issue-scale categories so that the ordering maximizes correct classification of responses. In two dimensions, OOC generates a cutting line for each response category of each item.^7^ For example, and for a given item, respondents who answer “agree” and “strongly agree” will appear on opposite sides of a cutting line. The end result of the procedure is a “spatial map” of respondent coordinates, to which cutting lines can be added as desired.

OOC has several advantages over factor analysis and ideal-point estimators. These are addressed at length by Hare, Liu, and Lupton (2018). Crucially, OOC does not call for *ex ante* identifying restrictions (e.g., fixing the coordinates of respondents and/or items, as in an item-response model). Rather, the procedure (in two dimensions) needs to know just one person “on the right” and one “to the north.” We automated these discretionary decisions with principal-components analysis.

One OOC metric tells us about dimensionality. This is the direction of the “normal vector,” to which a series of cutting lines run perpendicular. A normal vector that runs 45 degrees, for example, points to the northwest. Affirmative answers are conservative on both dimensions, and the item helps define both. If a normal vector runs 90 degrees, the item loads exclusively onto the second dimension. The same goes for a vector of –90 degrees.

Two more metrics tell us about model fit. One is the percent of responses correctly classified. More useful is the proportional reduction in error (PRE), as it weights the procedure’s performance by ease of the classification task. For example, if an overwhelming majority of respondents think that “elected officials should stop talking and take action” (a typical result; see Lavezzolo and Ramiro 2018, p. 275), we need no dimensional analysis to get most answers right. PRE runs from zero to one. OOC routines with opinion data turn up PREs from 0.2 to 0.6, which we take as a good fit (Hare, Liu, and Lupton 2018, pp. 69–70).

## Results

Results are reported below. The tables are sorted in descending order of the normal-vector angles, such that items at the tops and bottoms of the tables most strongly load onto the respective second dimension. In both cases, normal vectors for the items of interest run more vertically than those for all but two other items (taxes and racial fear, both CCES). Hence they anchor the second dimension.

**Figure 1.**
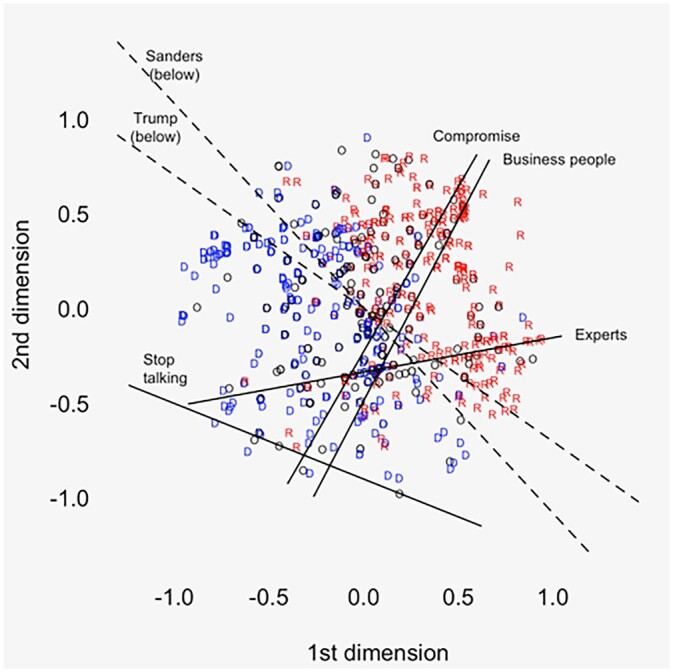
**OOC coordinates and cutting lines for “agree” (versus neutrality and disagreement) on the stealth-democracy items, CCES sample.** Dashed lines are estimated primary cleavages. “D” is Clinton preference, “R” is Trump preference, and “O” is other preference.

**Figure 2.**
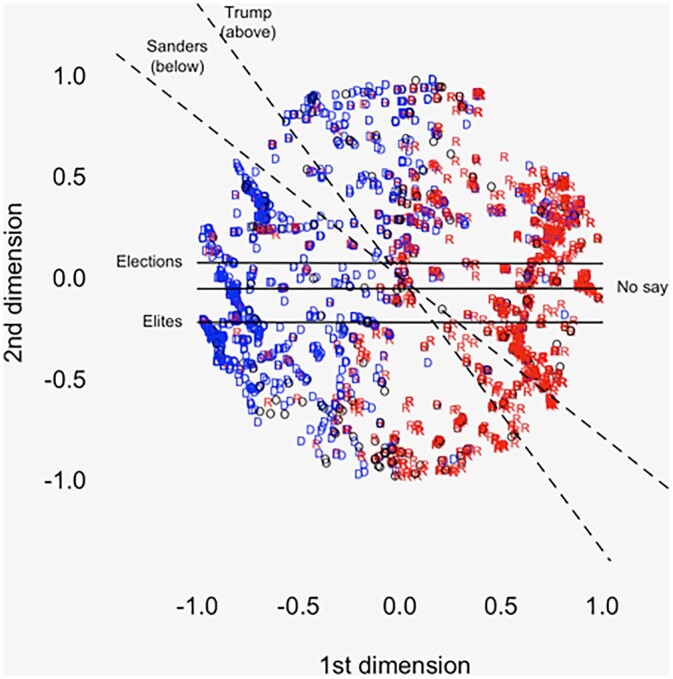
**OOC coordinates and cutting lines for “agree” (versus neutrality and disagreement) on the external-efficacy items, VSG sample.** Dashed lines are estimated primary cleavages. “D” is Clinton voter, “R” is Trump voter, and “O” is other/nonvoter.

The cutting lines for items of interest appear in figures 1 and 2 (CCES and VSG, respectively). Both figures clearly recover the party system: Clinton voters on the left, Trump voters on the right. The respondent coordinates are correlated with five-point ideology in a manner consistent with the first dimension tapping liberal-conservative.^8^

With respect to discontent, the VSG results are stark. All three items essentially divide the space into top and bottom halves. A slightly different picture emerges from CCES. Here, the line for “unelected experts” behaves as the external-efficacy items do in VSG. The “stop talking” line also runs horizontally, with nearly all respondents giving affirmative answers. But the other three items clearly tap both dimensions. This is consistent with recent work finding that “stealth democrats” tend to identify as conservative (Bengtsson and Mattila 2009; VanderMolen 2017; Medvic 2019) and/or vote for right-wing “populist” parties (Lavezzolo and Ramiro 2018).

How do results for other items differ from previous studies? Compared to the earlier analysis of VSG items (Santucci 2020), racial resentment, immigration, and capital punishment now load more strongly onto the first dimension. This underscores the role of political discontent in defining the second dimension.^9^

Turning to voting behavior, figures 1 and 2 include “cleavage lines” for Sanders and Trump voting in the 2016 primaries. These are estimated via probit (Schofield, Miller, and Martin 2003, p. 229)^10^ and give a *rough* view of the parties’ internal divisions.^11^ In CCES, the Trump line together with the “business” and “experts” lines bounds off a large part of the Trump primary electorate. Again, in VSG, the results are more stark. All five lines bound off much of the “insurgent” voting bloc in each party. Regression analysis in the Supplementary Material (sections IV and V) confirms the relationship with vote choice. Namely, attitudes toward “elites” predict both Sanders and Trump voting (VSG), the “no say” item predicts Sanders voting (VSG), and three “stealth” items predict Trump voting (business, experts, stop talking, all CCES).

**Table 1.** Fit statistics and direction of agreement (normal-vector angle) for the CCES sample

Item	PRE1	PRE2	PRE3	%CC1	%CC2	%CC3	Direction 2D
Stop talking	0.09	0.01	0.66	49.80	46.50	81.60	68.24
Increase prison sentences	0.24	0.02	–0.03	87.00	83.30	82.40	65.28
Trade (Trans-Pacific Partnership)	0.37	0.46	0.55	72.50	76.30	80.30	43.76
Regulate emissions	0.59	0.62	0.75	86.70	87.90	91.90	33.49
Environment/jobs trade-off	0.65	0.62	0.71	85.60	84.40	88.00	30.42
Whites have advantages	0.58	0.70	0.71	59.10	71.90	71.80	30.04
Abortion	0.44	0.51	0.49	77.60	80.20	79.50	28.44
Deport illegal immigrants	0.57	0.55	0.65	82.00	81.20	85.60	26.78
Sales vs. income tax	0.32	0.35	0.38	65.80	67.90	68.80	25.73
Gay marriage	0.43	0.45	0.45	80.80	81.60	81.70	25.65
Angry racism exists	0.44	0.45	0.53	66.70	69.50	71.40	25.02
Mandatory minimums	0.32	0.29	0.40	76.30	75.40	79.30	17.93
Legalize “dreamers”	0.50	0.61	0.59	76.70	81.70	80.90	17.09
Health care spending (state)	0.47	0.55	0.55	58.60	66.30	63.30	15.33
Repeal Affordable Care Act	0.72	0.73	0.78	87.50	87.70	89.90	12.13
Increase visas	0.03	0.17	–0.03	83.60	86.00	82.60	3.98
Racial problems are rare	0.41	0.49	0.59	57.90	61.80	70.30	2.92
Business people	0.52	0.68	0.68	59.90	73.60	72.30	–27.56
Compromise	0.32	0.47	0.55	54.10	63.70	68.90	–29.91
Raise taxes	0.16	0.42	0.54	48.20	66.40	73.00	–62.89
Fear other races	0.17	0.50	0.56	41.10	66.60	68.80	–71.80
Independent experts	0.16	0.58	0.78	42.70	69.60	84.30	–79.71

Note.—“PRE” is proportional reduction in error. “%CC” is percent of responses correctly classified. The integer after each refers to the number of dimensions in the respective routine.

**Table 2.** Fit statistics and direction of agreement (normal-vector angle) for the VSG sample

Item	PRE1	PRE2	PRE3	%CC1	%CC2	%CC3	Direction 2D
Foreign trade	0.19	0.16	0.01	74.90	81.70	78.50	66.95
Ease of immigration	0.34	0.45	0.76	25.30	57.20	78.80	58.48
Other minorities overcame	0.65	0.74	0.80	33.70	81.30	85.30	41.22
Blacks should try harder	0.71	0.72	0.79	32.80	77.40	83.00	37.17
Generations of slavery	0.69	0.75	0.76	31.00	79.40	79.80	30.37
Death penalty frequency	0.61	0.66	0.73	45.80	76.40	82.00	29.17
Blacks have gotten less	0.65	0.70	0.78	32.70	78.00	84.00	27.66
Death penalty	0.59	0.64	0.66	62.70	88.00	88.70	22.06
Immigrants make contribution	0.72	0.75	0.81	45.20	81.50	86.90	–1.80
Immigrant legalization path	0.63	0.65	0.70	55.50	86.30	88.20	–3.05
Affirmative action	0.72	0.76	0.76	56.50	89.50	89.80	–12.65
Gay marriage	0.58	0.58	0.64	55.20	83.30	85.70	–15.35
Health reform bill	0.86	0.89	0.89	49.30	92.60	92.80	–15.91
Abortion	0.36	0.50	0.55	48.10	75.70	77.90	–16.03
Regulation of business	0.67	0.75	0.73	40.70	82.60	81.90	–22.97
Global warming denial	0.62	0.77	0.83	37.60	81.90	86.00	–24.65
Universal healthcare	0.83	0.85	0.86	55.30	92.30	93.20	–26.36
Humans cause warming	0.65	0.74	0.80	60.50	92.60	94.10	–30.36
Higher taxes >$200 k	0.59	0.76	0.79	60.50	92.10	93.10	–39.51
No say	0.07	0.42	0.41	40.60	65.70	65.60	–82.63
Elites don't understand	0.11	0.20	0.50	42.10	52.80	71.80	–83.26
Elections don't matter	0.05	0.29	0.22	45.20	59.50	55.90	–88.15

Note.—“PRE” is proportional reduction in error. “%CC” is percent of responses correctly classified. The integer after each refers to the number of dimensions in the respective routine.

## Conclusion

This note has explored the structure of American political discontent as of 2016. It did so by acknowledging the two-dimensional structure of public opinion and by projecting relevant indicators onto that structure. A few of these indicators have more conservative content than others, but all of them point to a second dimension anchored by political discontent. Here, we acknowledge work by Uscinski et al. (2021) that recovers an “anti-establishment” dimension and connects it to Trump and Sanders support in 2020.

Discontent is related to but distinct from “populism.” With others, we view discontent as a *latent* dimension that can be tapped with many measures (Jennings et al. 2017; Hibbing et al. 2021). Populism refers to its political mobilization, which, again, can happen on the “left” or the “right” (Mudde and Rovira Kaltwasser 2017). So far, the conversation about populism has tended to focus on other countries—mainly in Europe and Latin America. There, the mobilization of discontent typically involves new parties. Because the United States has just two viable parties, discontent finds its expression within them (Dyck, Pearson-Merkowitz, and Coates 2018). More generally, we argue (and hope to have shown) that a focus on one dimension is not sufficient for understanding mass behavior in modern American politics.

## Data Availability Statement

REPLICATION DATA AND DOCUMENTATION are available at https://osf.io/vb5wp.

## Supplementary Material

SUPPLEMENTARY MATERIAL may be found in the online version of this article: https://doi.org/10.1093/poq/nfac009.

## Supplementary Material

nfac009_Supplementary_Data

## Footnotes

1
Uscinski et al. (2021) include conspiracy thinking in their discontent battery. Their study differs most starkly from ours in using four nonpolicy items to tap liberal-conservative.

2 The 2016 CCES data and methodology report are publicly available here: https://doi.org/10.7910/DVN/M72A0Q.

3 The 2016 VSG data and methodology report are publicly available here: https://www.voterstudygroup.org/publication/2016-voter-survey.

4 The first dimension captured, in descending order: healthcare, taxation, the environment, gay marriage, affirmative action, and abortion. The second dimension captured racial resentment, immigration, and capital punishment.

5 The question asked about how to deal with the federal budget deficit. Respondents were asked to rank three options: cutting taxes, cutting domestic spending, and cutting defense spending.

6 Other differences are when the surveys fielded, then that CCES emphasizes policy, whereas VSG emphasizes nationally salient attitudes.

7 Technically, for a c-category item, it generates c-1 cutting lines.

8 In CCES, first-dimension coordinates correlate with five-point ideology at 0.49. Second-dimension coordinates correlate at 0.02. These figures for VSG are 0.64 and 0.02.

9 Several first-dimension issues from the Santucci (2020) study were second-dimension issues in earlier work, that is, abortion, the environment, and same-sex marriage (Treier and Hillygus 2009, p. 686). This is consistent with “conflict extension,” or the over-time uptake of new issues by the party system (Layman and Carsey 2002).

10 The reference category in each model is a same-party primary voter who chose someone else. The respective variables are: CC16_328 (CCES primary vote), CC16_364c (CCES presidential preference), pp_demprim16_2016 (VSG Democratic primary vote), pp_repprim16_2016 (VSG Republican primary vote), and presvote16post_2016 (VSG presidential vote).

11 Note that the angles of the primary cleavages in both data sets are reversed, as is that of the overall cleavage between general-election voting blocs. This may be due to differences between the sets of items (e.g., how racism is measured), the time at which the surveys were taken (August versus December), or the fact that the CCES sample includes the stealth-democracy items (three of which predict Trump support in the primaries). Further, we stress that these cleavage lines are estimated, that is, they do not perfectly separate voting blocs.

## Notes

The authors thank discussants and audience members at the 2018 Annual Meetings of the Midwest and American Political Science Associations. The 2016 Cooperative Congressional Election Study data were collected by YouGov with support from numerous sources. The 2016 Voter Study Group data were collected by YouGov with support from the Democracy Fund.

## References

[nfac009-B1] Akkerman Agnes , Mudde Cas, Zaslove Andrej. 2014. “How Populist Are the People? Measuring Populist Attitudes in Voters.” Comparative Political Studies 47:1324–53. doi:10.1177/0010414013512600.

[nfac009-B2] Axelrod Robert. 1967. “The Structure of Public Opinion on Policy Issues.” Public Opinion Quarterly 31:51–60. doi:10.1086/267481.

[nfac009-B3] Bengtsson Åsa , Mattila Mikko. 2009. “Direct Democracy and Its Critics: Support for Direct and ‘Stealth’ Democracy in Finland.” West European Politics 32:1031–48. doi:10.1080/01402380903065256.

[nfac009-B4] Buisseret Peter , Richard Van Weelden. 2020. “Crashing the Party? Elites, Outsiders, and Elections.” American Journal of Political Science 64:356–70. doi:10.1111/ajps.12457.

[nfac009-B5] Coffé Hilde , Ank Michels. 2014. “Education and support for representative, direct and stealth democracy.” Electoral Studies 35:1–11. doi:10.1016/j.electstud.2014.03.006.

[nfac009-B6] Craig Stephen C. 1980. “The mobilization of political discontent.” Political Behavior 2:189–209. doi:10.1007/BF00989890

[nfac009-B7] Craig Stephen C. , Niemi Richard G., Silver Glenn E.. 1990. “Political Efficacy and Trust: A Report on the NES Pilot Study Items.” Political Behavior 12:289–314. doi:10.1007/BF00992337.

[nfac009-B8] Democracy Fund Voter Study Group. August 28, 2017. Views of the Electorate Research Survey, December 2016. [Computer File] Release 1. Washington, DC: Democracy Fund Voter Study Group [producer], available at https://www.voterstudygroup.org/.

[nfac009-B9] DeSante Christopher D. , Smith Candis Watts. 2020. “Fear, Institutionalized Racism, and Empathy: The Underlying Dimensions of Whites’ Racial Attitudes.” PS: Political Science & Politics 53:639–45. doi:10.1017/S1049096520000414.

[nfac009-B10] Drutman Lee. 2016. “What Paul Ryan’s Budget Woes Tell Us about the Continued Crack-Up of the Republican Party.” *Vox.com*, April 11. Available at https://www.vox.com/polyarchy/2016/4/11/11406228/ryan-budget-gop-crackup.

[nfac009-B11] Dyck Joshua J. , Pearson-Merkowitz Shanna, Coates Michael. 2018. “Primary Distrust: Political Distrust and Support for the Insurgent Candidacies of Donald Trump and Bernie Sanders in the 2016 Primary.” PS: Political Science & Politics 51:351–57. doi:10.1017/S1049096517002505.

[nfac009-B12] Geurkink Bram , Zaslove Andrej, Sluiter Roderick, Jacobs Kristof. 2020. “Populist Attitudes, Political Trust, and External Political Efficacy: Old Wine in New Bottles?” Political Studies 68:247–67. doi:10.1177/0032321719842768.

[nfac009-B13] Gronke Paul. 2019. “CCES 2016, Team Module of Reed College (RC).” Available at 10.7910/DVN/M72A0Q, Harvard Dataverse, V1, UNF:6:4Y9SWLRWxUduUIUB34u9ZA== [fileUNF].

[nfac009-B14] Hare Christopher , Liu Tzu-Ping, Lupton Robert N.. 2018. “What Ordered Optimal Classification Reveals about Ideological Structure, Cleavages, and Polarization in the American Mass Public.” Public Choice 176:57–78. doi:10.1007/s11127-018-0540-6.

[nfac009-B15] Hibbing John R. , Theiss-Morse Elizabeth. 2002. Stealth Democracy: Americans’ Beliefs about How Government Should Work. New York: Cambridge University Press.

[nfac009-B16] Hibbing John R. , Theiss-Morse Elizabeth, Hibbing Matthew V., Fortunato David. 2021. “Who Do the People Want to Govern?” Party Politics? 135406882110500. doi:10.1177/13540688211050064.

[nfac009-B1002] Jennings, Will, Clarke Nick, Moss Jonathan, and Stoker Gerry. 2017. “The Decline in Diffuse Support for National Politics: The Long View on Political Discontent in Britain.” *Public Opinion Quarterly* 81:748–758. doi:10.1093/poq/nfx020. 10.1093/poq/nfx020 PMC5927329 29731522

[nfac009-B1003] Kinder, Donald R., and Kalmoe Nathan P. 2017. Neither Liberal nor Conservative: Ideological Innocence in the American Public. Chicago: University of Chicago Press.

[nfac009-B1001] Lavezzolo, Sebastián, and Ramiro Luis. 2018. “Stealth Democracy and the Support for New and Challenger Parties.” *European Political Science Review* 10:267–289. doi:10.1017/S1755773917000108.

[nfac009-B17] Layman Geoffrey C. , Carsey Thomas M.. 2002. “Party Polarization and ‘Conflict Extension’ in the American Electorate.” American Journal of Political Science 46:786–802. doi:10.2307/3088434.

[nfac009-B18] Lipset Seymour M. 1959. “Democracy and Working-Class Authoritarianism.” American Sociological Review 24:482–501. doi:10.2307/2089536.

[nfac009-B19] Medvic Steven. 2019. “Explaining Support for Stealth Democracy.” Representation 55:1–19. doi:10.1080/00344893.2019.1581076.

[nfac009-B20] Menchaca Marcos. 2021. “Are Americans Polarized on Issue Dimensions?” Journal of Elections, Public Opinion and Parties 1. Early version. doi:10.1080/17457289.2021.1910954.

[nfac009-B21] Mudde Cas , Rovira Kaltwasser Cristobal. 2017. Populism: A Very Short Introduction. New York: Oxford University Press.

[nfac009-B22] Rooduijn Matthijs , van der Brug Wouter, de Lange Sarah L.. 2016. “Expressing or Fueling Discontent? The Relationship Between Populist Voting and Political Discontent.” Electoral Studies 43:32–40. doi:10.1016/j.electstud.2016.04.006.

[nfac009-B23] Santucci Jack. 2020. “Did the Party System Change from 2012–2016?” Journal of Elections, Public Opinion and Parties 1. doi:10.1080/17457289.2020.1794884.

[nfac009-B24] Schofield Norman , Miller Gary, Martin Andrew. 2003. “Critical Elections and Political Realignments in the USA: 1860–2000.” Political Studies 51:217–40. doi:10.1111/1467-9248.00421.

[nfac009-B25] Spruyt Bram , Keppens Gil, Van Droogenbroeck Filip. 2016. “Who Supports Populism and What Attracts People to It?” Political Research Quarterly 69:335–46. doi:10.1177/1065912916639138.

[nfac009-B26] Treier Shawn , Hillygus D. Sunshine. 2009. “The Nature of Political Ideology in the Contemporary Electorate.” Public Opinion Quarterly 73:679–703. doi:10.1093/poq/nfp067.

[nfac009-B27] Uscinski Joseph E. , Enders Adam M., Seelig Michelle I., Klofstad Casey A., Funchion John R., Everett Caleb, Wuchty Stefan, Premaratne Kamal, Murthi Manohar N.. 2021. “American Politics in Two Dimensions: Ideological Identities versus Anti-Establishment Orientations.” American Journal of Political Science 65:877–95. doi:10.1111/ajps.12616.

[nfac009-B28] VanderMolen Kathryn. 2017. “Stealth Democracy Revisited: Reconsidering Preferences for Less Visible Government.” Political Research Quarterly 70:687–98. doi:10.1177/1065912917712478.

