# Supplementary material for: The Structure of American Political Discontent
Source: Public Opin Q. 2022 Apr 29;86(2):381–92. doi: 10.1093/poq/nfac009 (PMC9186374; doi:10.1093/poq/nfac009)
Supplement: nfac009_Supplementary_Data [file nfac009_supplementary_data.pdf]

Supplementary Material

The Structure of American Political Discontent

Public Opinion Quarterly

Jack Santucci
Drexel University
jack.santucci@gmail.com

Joshua J. Dyck
University of Massachusetts Lowell
joshua_dyck@uml.edu

I. AAPOR Ethics Data Disclosure

II. Full Question Wording of Items Included, CCES ROC Module (2016)

III. Full Question Wording of Items Included, Voter Study Group (2016)

IV. Primary Regression Results (CCES)

V. Primary Regression Results (VSG)

I. AAPOR Ethics Data Disclosure

Appendix Table A1. AAPOR Ethics Data Disclosure

	CCES Module (ROC - 2016)	VSG (2016)
Funding Disclosure	None, the data were accessed via public download here	None, the data were accessed via public download here
Question wording for all survey items	See Appendix A2	See Appendix A2
Population & Geography	American adults 18 and over	American adults 18 and over
Sampling Frame	US Citizens from the American Community Survey	US Citizens from the American Community Survey
Sample design & method of data collection	From the 2016 CCES Codebook:<br><br>"The 2016 CCES survey was conducted over the Internet by YouGov. The Common Content was asked of 64,600 adults interviewed in October 2016 (for pre election data), and in November 2016 (for post election data). The sampling method uses YouGov's matched random sample methodology.<br><br>Sample matching is a methodology for selection of "representative" samples from nonrandomly selected pools of respondents. It is ideally suited for Web access panels, but could also be used for other types of surveys, such as phone surveys. Sample matching starts with an enumeration of the target population. For general population studies, the target population is all adults, and can be enumerated through the use of the decennial Census or a high quality survey, such as the American Community Survey. In other contexts, this is known as the sampling frame, though, unlike conventional sampling, the sample is not drawn from the frame. Traditional sampling, then, selects	From the VSG methodology report:<br><br>"The Views of the Electorate Research (VOTER) Survey was conducted by the survey firm YouGov. In total, 8,000 adults (age 18+) with internet access took the survey on-line between November 29 and December 29, 2016. The reported margin of error is plus or minus 2.2%. YouGov also supplied measures of primary voting behavior from the end of the primary period (July 2016), when these respondents had been contacted as part of a different survey project.<br><br>These respondents were originally interviewed by YouGov in 2011-2012 as part of the 2012 Cooperative Campaign Analysis Project (CCAP). In that survey, 45,000 respondents were first interviewed in December 2011 and were interviewed a second time in one of the 45 weekly surveys between January 1 and November 8, 2012. After the November election, 35,408 respondents were interviewed a third time. For this survey 11,168 panelists from 2012 CCAP were invited to respond

	individuals from the sampling frame at random for participation in the study. This may not be feasible or economical as the contact information, especially email addresses, is not available for all individuals in the frame and refusals to participate increase the costs of sampling in this way.<br><br>Sample selection using the matching methodology is a two-stage process. First, a random sample is drawn from the target population. We call this sample the target sample. Details on how the target sample is drawn are provided below, but the essential idea is that this sample is a true probability sample and thus representative of the frame from which it was drawn. However, YouGov is not able to contact these individuals directly. Therefore, the second step is that for each member of the target sample, we select one or more matching members from our pool of opt-in respondents. This is called the matched sample. Matching is accomplished using a large set of variables that are available in consumer and voter databases for both the target population and the opt-in panel.<br><br>The purpose of matching is to find an available respondent who is as similar as possible to the selected member of the target sample. The result is a sample of respondents who have the same measured characteristics as the target sample. Under certain conditions, described below, the matched sample will have similar properties to a true	and 8,637 of them (77%) completed the 2016 survey.<br><br>The 2012 CCAP was constructed using YouGov's sample matching procedure. A stratified sample is drawn from YouGov's panel, which consists of people who have agreed to take occasional surveys. The strata are defined by the combination of age, gender, race, and education, and each stratum is sampled in proportion to its size in the U.S. population. Then, each element of this sample is matched to a synthetic sampling frame that is constructed from the U.S. Census Bureau's American Community Survey, the Current Population Survey Voting and Registration Supplement, and other databases. The matching procedure finds the observation in the sample from YouGov's panel that most closely matches each observation in the synthetic sampling frame on a set of demographic characteristics. The resulting sample is then weighted by a set of demographic and non-demographic variables (in the dataset, this is the variable "weight")."

	random sample. That is, the matched sample mimics the characteristics of the target sample. It is, as far as we can tell, representative of the target population (because it is similar to the target sample).<br><br>When choosing the matched sample, it is necessary to find the closest matching respondent in the panel of opt-ins to each member of the target sample. Various types of matching could be employed: exact matching, propensity score matching, and proximity matching."<br><br>The data for this project come from one of the 60 team modules that make up the 2016 CCES. All parts are collected using the same process and each team module is produces a nationally representative dataset of 1000 Americans 18 and older.	
Date of data collection	Pre: 9/28/16-11/7/16<br>Post: 11/9/16-12/14/16	11/29/2016-12/29/2016
Response Rate	RR1=.419<br>CC1=.848<br>Full details here (p. 14)	RR1=.178<br>CC1=.716<br>Full details are available here
Sample Sizes	N=1000	N=8000
Sampling Error	+/-4.3%<br>(adjusted for design effects)	+/-2.2%<br>(adjusted for design effects)
Were analyses adjusted for design effects/sample weights?	Optimal classification does not incorporate survey weights. The weights were not used in any of the analyses in this research note.	Optimal classification does not incorporate survey weights. The weights were not used in any of the analyses in this research note.

II. Full Question Wording of Items Included, CCES ROC Module (2016)

Immigration (3 questions): What do you think the U.S. government should do about immigration? Select all that apply. (response options: selected; not selected)

CC16_331_3: Grant legal status to people who were brought to the US illegally as children, but who have graduated from a U.S. high school
CC16_331_6: Increase the number of visas for overseas workers to work in the U.S.
CC16_331_7: Identify and deport illegal immigrants

Abortion/Criminal Justice Reform (3 questions): Do you support or oppose each of the following proposals? (response options: Support; Oppose)

CC16_332a: Always allow a woman to obtain an abortion as a matter of choice
CC16_334a: Eliminate mandatory minimum sentences for non-violent drug offenders
CC16_334d: Increase prison sentences for felons who have already committed two or more serious or violent crimes

CC16_335: Do you favor or oppose allowing gays and lesbians to marry legally? (response options: Favor; Oppose)

The federal budget deficit is approximately $1 trillion this year. If the Congress were to balance the budget it would have to consider cutting defense spending, cutting domestic spending (such as Medicare and Social Security), or raising taxes to cover the deficit. Please rank the options below from what would you most prefer that Congress do to what you would least prefer they do." (response options: 1, 2 or 3 -- ranking)

CC16_337_3: Raise Taxes (use rank ordering scale 1-3)

Congress considers many issues. If you were in Congress would you vote FOR or AGAINST each of the following?

CC16_351b: Trans-Pacific Partnership Act Free trade agreement among 12 Pacific nations (Australia, Brunei, Canada, Chile, Japan, Malaysia, Mexico, New Zealand, Peru, Singapore, and the US).

Race/Racism (4 questions): To what extent do you agree or disagree with the following statements? (response options: Strongly disagree; Somewhat disagree; Neither agree nor disagree; Somewhat agree; Strongly agree)

CC16_422c: I am angry that racism exists
CC16_422d: White people in the U.S. have certain advantages because of the color of their skin
CC16_422e: I often find myself fearful of people of other races
CC16_422f: Racial problems in the U.S. are rare, isolated situations

CC16_416r: If the state had to raise taxes, what share of the tax increase should come from increased income taxes and what share from increased sales taxes? Choose a point along the scale from 100

Stealth Democracy (4 questions): People have different opinions about politics and government. Do you agree or disagree with the following statements? (response options: Strongly Agree; Agree; Neither Agree nor Disagree; Disagree; Strongly Disagree)

> RCO340: Elected officials would help the country more if they would stop talking and just take action on important problems
> RCO341: What people call ‘compromise’ in politics is really just selling out on one’s principles
> RCO342: Our government would run better if decisions were left up to successful business people
> RCO343: Our government would run better if decisions were left up to non-elected, independent experts rather than politicians or the people

III. Full Question Wording of Items Included, Voter Study Group (2016)

abortview3_2016: Do you think abortion should be... (response options: Legal in all cases; Legal/Illegal in some; Illegal in all cases)

immi_contribution_2016: Overall, do you think illegal immigrants make a contribution to American society or are a drain? (response options: Mostly make a contribution; Neither; Mostly a drain)

immi_naturalize_2016: Do you favor or oppose providing a legal way for illegal immigrants already in the United States to become U.S. citizens? (response options: Favor; Oppose)

immi_makedifficult_2016: Do you think it should be easier or harder for foreigners to immigrate to the US legally than it is currently? (response options: Much easier; Slightly easier; No change: Slightly harder: Much harder)

gaymar_2016: Do you favor or oppose allowing gays and lesbians to marry legally? (response options: Favor; Oppose)

deathpen_2016: Are you in favor or opposed to the death penalty for persons convicted of murder? (response options: Favor the death penalty; Opposed to the death penalty)

deathpenfreq_2016: Do you think the death penalty is imposed too often or not often enough? (response options: Too often; About right; Not often enough)

univhealthcov_2016: Do you think it is the responsibility of the federal government to see to it that everyone has health care coverage? (response options: Yes; No)

healthreformbill_2016: Do you think the health care reform bill should be expanded, kept the same, or repealed? (response options: Expanded; Kept the same; Repealed)

envwarm_2016: Some people say that global temperatures have been going up slowly over the past 100 years - the phenomenon called "global warming." Do you think that global warming is happening? (response options: Definitely is happening; Probably is happening; Probably is not happening; Definitely is not happening)

envpoll2_2016: Do you think global warming has been caused by pollution from human activities (such as emissions from cars and factories) or by natural causes? (response options: Pollution from human activities; Natural causes not related to human activities)

affirmact_gen_2016: Do you generally favor or oppose affirmative action programs for women and racial minorities? (response options: Favor; Oppose)

taxdoug_2016: Do you favor raising taxes on families with incomes over $200,000 per year? (response options: Yes; No)

govt_reg_2016: In general, do you think there is too much or too little regulation of business by the government? (response options: Too much; About the right amount; Too little)

tradepolicy_2016: Do you favor or oppose increasing trade with other nations? (response options: Favor; Oppose)

Racial Issues (4 items): Here are a few statements about race in America. Please tell uswhether you agree or disagree with each statement. (response options: Strongly agree; Agree; Somewhat disagree; Disagree)

race_deservemore_2016: Over the past few years, Blacks have gotten less than they deserve
race_overcome_2016: Other minorities overcame prejudice and worked their way up, Blacks should do the same without any special favors
race_tryharder_2016: It's really a matter of some people not trying hard enough; if Blacks would only try harder they could be just as well off as Whites
race_slave_2016: Generations of slavery and discrimination have created conditions that make it difficult for Blacks to work their way out of the lower class

Efficacy Battery (3 items): Please tell us whether you agree or disagree with each of the following statements: (response options: Strongly agree; Agree; Disagree; Strongly disagree)

RIGGED_SYSTEM_1_2016: Elections today don’t matter; things stay the same no matter who we vote in.
RIGGED_SYSTEM_5_2016: People like me don't have any say in what the government does.
RIGGED_SYSTEM_6_2016: Elites in this country don't understand the problems I am facing.

IV. Primary Regression Results (CCES)

Linear probability models of 2016 primary vote, CCES/ROC data. All predictors are rescaled to run [0,1]. Nonvoters are excluded.

	**Sanders**	**Sanders**	**Sanders**	**Sanders**	**Trump**	**Trump**	**Trump**	**Trump**
(Intercept)	0.74^{***}	0.77^{***}	0.70^{***}	0.66^{***}	0.52^{*}	0.77^{***}	0.75^{***}	0.63^{***}
	(0.14)	(0.10)	(0.09)	(0.10)	(0.21)	(0.19)	(0.18)	(0.19)
Ideology	-0.45^{***}	-0.45^{***}	-0.45^{***}	-0.48^{***}	-0.08	-0.17	-0.14	-0.04
	(0.11)	(0.12)	(0.12)	(0.12)	(0.17)	(0.17)	(0.17)	(0.17)
White (0,1)	0.14^{*}	0.12^{*}	0.14^{*}	0.14^{**}	0.08	0.12	0.08	0.11
	(0.06)	(0.06)	(0.06)	(0.06)	(0.10)	(0.10)	(0.10)	(0.10)
Education	-0.10	-0.11	-0.10	-0.11	-0.38^{***}	-0.42^{***}	-0.46^{***}	-0.41^{***}
	(0.09)	(0.09)	(0.09)	(0.09)	(0.11)	(0.11)	(0.11)	(0.11)
Woman (0,1)	-0.09	-0.08	-0.08	-0.09	0.05	0.08	0.09	0.07
	(0.05)	(0.05)	(0.05)	(0.05)	(0.06)	(0.06)	(0.06)	(0.06)
Family Income	-0.15	-0.15	-0.15	-0.14	0.03	0.01	0.03	0.02
	(0.11)	(0.11)	(0.11)	(0.11)	(0.09)	(0.09)	(0.09)	(0.09)
Age 34-49	-0.16^{*}	-0.15^{*}	-0.16^{*}	-0.15	-0.03	-0.02	-0.06	0.00
	(0.08)	(0.08)	(0.08)	(0.08)	(0.11)	(0.11)	(0.11)	(0.10)
Age 50-64	-0.09	-0.10	-0.09	-0.07	-0.22^{*}	-0.20^{*}	-0.24^{*}	-0.18^{*}
	(0.07)	(0.07)	(0.07)	(0.07)	(0.09)	(0.09)	(0.09)	(0.09)
Age 65+	-0.33^{***}	-0.33^{***}	-0.33^{***}	-0.31^{***}	-0.25^{*}	-0.26^{*}	-0.29^{**}	-0.23^{*}
	(0.08)	(0.08)	(0.08)	(0.08)	(0.10)	(0.10)	(0.10)	(0.10)
Stealth Dem: Stop Talking	-0.05				0.39^{**}			
	(0.12)				(0.14)			
Stealth Dem: Compromise		-0.11				0.17		
		(0.09)				(0.13)		
Stealth Dem: Business			0.00				0.27^{*}	
			(0.10)				(0.11)	
Stealth Dem: Experts				0.09				0.29^{**}

				(0.10)				(0.10)
R^2	0.13	0.14	0.13	0.13	0.12	0.10	0.12	0.13
Adj. R^2	0.11	0.11	0.10	0.11	0.09	0.07	0.09	0.10
Num. obs.	321	321	322	322	256	258	256	260
***p < 0.001; **p < 0.01; *p < 0.05								

V. Primary Regression Results (VSG)

Linear probability models of 2016 primary vote, VSG data. All predictors are rescaled to run [0,1]. Nonvoters are excluded.

	**Sanders**	**Sanders**	**Sanders**	**Trump**	**Trump**	**Trump**
(Intercept)	0.40***	0.31***	0.21***	0.73***	0.68***	0.63***
	(0.04)	(0.04)	(0.04)	(0.06)	(0.06)	(0.06)
Ideology	-0.18***	-0.18***	-0.15***	-0.07	-0.11	-0.06
	(0.04)	(0.04)	(0.04)	(0.06)	(0.06)	(0.06)
White (0,1)	0.18***	0.17***	0.18***	-0.04	-0.03	-0.05
	(0.02)	(0.02)	(0.02)	(0.03)	(0.03)	(0.03)
Education	0.26***	0.27***	0.27***	-0.30***	-0.31***	-0.29***
	(0.03)	(0.03)	(0.03)	(0.04)	(0.04)	(0.04)
Woman (0,1)	-0.04*	-0.04*	-0.04*	0.02	0.02	0.03
	(0.02)	(0.02)	(0.02)	(0.02)	(0.02)	(0.02)
Family Income	-0.15**	-0.13**	-0.12*	-0.13*	-0.12*	-0.11*
	(0.05)	(0.05)	(0.05)	(0.05)	(0.05)	(0.05)
Age 34-49	-0.15***	-0.14***	-0.15***	0.07*	0.09**	0.03
	(0.03)	(0.03)	(0.03)	(0.03)	(0.03)	(0.03)
Age 50-64	-0.18***	-0.18***	-0.18***	0.05	0.07*	0.02
	(0.03)	(0.03)	(0.03)	(0.03)	(0.03)	(0.03)
Age 65+	-0.15***	-0.16***	-0.16***	0.07*	0.09**	0.04
	(0.03)	(0.03)	(0.03)	(0.03)	(0.03)	(0.03)
Ext. Efficacy: Elections	0.05			-0.02		
	(0.03)			(0.04)		
Ext. Efficacy: No Say		0.19***			0.06	
		(0.03)			(0.04)	
Ext. Efficacy: Elites			0.25***			0.15***
			(0.04)			(0.04)
R^2	0.11	0.12	0.13	0.05	0.06	0.05

Adj. R^2	0.11	0.12	0.13	0.05	0.05	0.05
Num. obs.	2524	2519	2522	2395	2394	2387
***p < 0.001; **p < 0.01; *p < 0.05						
